# Tuning the Physicochemical Properties of Nanostructured Materials through Advanced Preparation Methods

**DOI:** 10.3390/nano12060956

**Published:** 2022-03-14

**Authors:** John Vakros, George Avgouropoulos

**Affiliations:** 1 Department of Chemical Engineering, University of Patras, Caratheodory 1, University Campus, GR-26504 Patras, Greece; 2 Department of Materials Science, University of Patras, University Campus, GR-26504 Patras, Greece; geoavg@upatras.gr

Over the last few decades, nanotechnology has received a huge level of interest due to its extensive applications in various fields, including catalysis, electronics, optics, energy, and the environment. The main characteristic of nanostructured materials is their surface reactivity due to their active surface functional groups. The control of the size, shape, and nature of nanoparticles is strongly influenced by the synthetic route applied during the preparation step (i.e., hydrothermal, solvothermal, combustion, sol-gel). The design and controlled synthesis of advanced nanomaterials with unique properties make them highly attractive in these fields.

Today, nanotechnology, providing the capability to precisely manufacture small structures, is being promoted as a national policy in many countries. The progress of nanotechnology allows information and functions to be integrated in smaller spaces. There are two main categories for the synthesis and preparation of nanomaterials. The first deals with top-down methods, where the desired nanostructure is produced by decreasing the size of a larger substrate, while the second follows bottom-up approaches that assemble nanostructures from atoms and molecules. Nanomaterials can be classified into one-, two-, and three-dimensional materials [1]. The review by Ebina et al. [1] summarizes the research on different techniques developed to form one-, two-, and three-dimensional connected structures (CSs) of metal nanoclusters (NCs) through self-assembly. Metal nanoclusters (NCs) consisting from a few to about 100 metal atoms have gained significant interest due to their unique properties. Generally, these properties, in terms of surface reactivity, are different than the corresponding bulk metal. This is important, since the NCs are of an easy-to-handle size. Connected structures of 1D, 2D, and 3D can be synthesized through newly designed synthetic routes, which determine their geometrical structures and physical/chemical properties. The 1D, 2D, and 3D CSs are discussed in detail in the first sections of this review article and the authors give a brief outlook on their future.

An important class of materials for optical applications is semiconductor quantum dots (QDs) [2]. They have strong quantum confinement effects, as the size of QDs is close to the Bohr radius of the exciton. Among other QDs, PbS QDs is a representative zero-dimensional material. Compared to 1D and 2D nanomaterials, PbS QDs possess strong absorption and an adjustable bandgap in the near IR region. In the contribution of L. Yun and W. Zhao [2], fiber-based PbS QDs were used as saturable absorbers and tested for dual-wavelength ultrafast pulse generation. Specifically, PbS QDs, fabricated via a modified hot-injection method, have the advantages of fast relaxation time, wide bandwidth, large modulation depth, and thermal damage. With the introduction of PbS QDs into an erbium-doped fiber laser, the laser can simultaneously generate dual-wavelength conventional solitons with central wavelengths of 1532 and 1559 nm, with 3 dB bandwidths of 2.8 and 2.5 nm, respectively, and it can be adopted as a broadband SA for application in pulsed lasers.

The applications of nanoparticles are almost unlimited; for example, ceramic nanoparticles can be used as a reinforcement of lightweight alloys. In a recently published work [3], yttria is widely used in the ceramic technology. Specifically, solution combustion synthesis (SCS) was applied for the preparation of nanoyttria. This technique is based on the high-energy reaction between metal nitrates (yttrium nitrate, in this case) and a reducing agent such as glycine, leading to well-formed nanoparticles. The properties of yttria nanopowder were studied by several physicochemical techniques, including differential thermal analysis coupled with FT-IR spectrometry, for the online SCS monitoring, Scanning Electron Microscopy, measurements of their specific surface area with BET method, and particle size distribution. Interestingly, the obtained powders showed nanoscale structures after calcination at 1100 °C. The obtained nanopowder showed very high sintering activity as the shrinkage onset was detected at a temperature of about 1150 °C.

A new method was applied for the preparation of yttrium-doped barium cerate (BCY15) [4]. This material was used as an anode ceramic matrix for synthesis of the Ni-based cermet anode in a proton-conducting solid oxide fuel cell (pSOFC). Although SOFCs are very promising for clean energy production and have many advantages, they are difficult to find commercial applications for, due to the fact that their operation temperature is significantly high—between 800–1000 °C with a commercial goal of 600–700 °C. SOFCs require a reduction step for the supported metal, usually Ni. A modification on the preparation route with the application of hydrazine wet-chemical synthesis can be a low-cost alternative. This method promotes ‘in situ’ introduction of metallic Ni particles in the BCY15 matrix. Two different Ni/BCY15 cermets were synthesized using either water or ethylene glycol (EG). The samples were characterized using a variety of physicochemical techniques and resulted in a more active and stable Ni cermet with well-dispersed nanoscale metal Ni particles, due to stronger interactions between the Ni and BCY15 matrix. These factors contributed to better performance, higher stability and lower degradation rate during operation of a pSOFC.

The physicochemical properties of nanomaterials are greatly influenced by their dimensions. Thus, the dispersion media are very important. In the absence of a dispersion medium, the nanoparticles tend to agglomerate and lose their interesting properties. The aggregation of nanoparticles depends on both their characteristics and those of the different media. The dispersion medium can be a solid support, in the case of solid catalyst, a liquid medium, or even cell culture media; thus, the effect of the various biological dispersion media on the state of aggregation of the nanoparticles has been extensively investigated in the literature and reviewed in [5]. Generally, nanomaterials interact with the surrounding environment and an interface is formed, whose properties depend on the physicochemical interactions and on colloidal forces. Parameters such as size, shape, surface chemistry, surface charge of the nanomaterials, and properties of the dispersion medium affect the behavior in a test medium. The relationship between the nanomaterials’ properties and their practical use is defined as Functionality. It is important to understand this relationship for the safe use of these nanomaterials, since it can play an important role on the safe design of manufactured nanomaterials (MNMs), thus, reducing the possible health and environmental risks early in the innovation process, where the functionality of a nanomaterial and its toxicity/safety will be taken into account in an integrated way. In this mini review, the authors attempt to identify the key parameters of the nanomaterials and establish a relationship between those and the main functionalities of the nanomaterials. Finally, the review aims to contribute to the decision tree strategy for the optimum design of safe nanomaterials.

In the field of catalysis, the preparation of the supported catalyst is, in many cases, crucial for the performance of the catalyst. The main physicochemical characteristics of the supported catalyst can be defined by the preparation route and the composition of the active phase [6]. For example, monoclinic zirconia-supported platinum (Pt/m-ZrO_2_) catalysts are active for the ethanol steam-reforming reaction. The reaction proceeds via ethanol dissociation to ethoxy species. The next step is oxidative dehydrogenation to acetate followed by acetate decomposition. This last step strongly depends on the catalysts’ composition. The desired step is the decarboxylation pathway, which tends to produce higher overall hydrogen selectivity, and can be promoted with the addition of alkali metal ions in the catalyst. Indeed, it was found that high loadings of K^+^ or Rb^+^ ions can promote hydrogen production, while for catalysts which are undoped or doped with low alkali loadings, decarbonylation is the preferable route. Thus, the overall hydrogen selectivity is significantly different between the two cases. Detailed studies with in situ diffuse reflectance infrared Fourier transform spectroscopy (DRIFTS) and the temperature-programmed reaction of ethanol steam reforming show that alkali doping promotes forward acetate decomposition while exposed metallic sites tend to facilitate decarbonylation [6].

Ceria (CeO_2_) is among the most extensively reducible supports that have been studied. Nanoceria exhibits interesting properties, among them high capacity to store and release oxygen, different surface ratio of Ce(III)/Ce(IV), bulk oxygen vacancies, and redox properties. These unique properties find significant applications in the field of catalysis. Especially when ceria is used as support for deposition of atomically dispersed Au, the corresponding catalyst exhibits superb performance in Water Gas Shift reaction (WGS). In this contribution from Prof. Tabakova’s group [7], layered double hydroxides NiAl (NiAl LDH) prepared via co-precipitation method were used as support for Au clusters, resulting in a catalyst with significant activity. By direct deposition of ceria on NiAl LDH and precipitation with NaOH, the authors were able to obtain the CeO_2_ phase and to preserve the NiAl layered structure by avoiding the calcination treatment. Using the deposition–precipitation method, Au was deposited in the modified support. The WGS performance of Au/NiAl catalysts was significantly affected by the addition of CeO_2_, as the CO conversion increased from 83.4% to 98.8% for the modified catalyst.

Hybrid biochar-ceria nanomaterials were synthesized using biochar (BC) from spent malt rootlets used as the template [8]. These hybrid materials were tested for the activation of persulfates (SPS) and subsequent degradation of sulfamethoxazole (SMX). Using wet impregnation, a simple preparation method, cerium nitrate was deposited on BC, and by regulating the calcination temperature, a tuning in the content of BC was achieved for the hybrid materials. The CeO_2_-BC hybrid materials were characterized, and it was found that calcination temperature affects the biochar content and the physicochemical properties of the hybrid materials. The most active materials were obtained with calcination temperatures of 300–350 °C, exhibiting high specific surface area, intense interactions between CeO_2_ and BC, and could degrade 500 μg/L SMX at about 60% within 2 h. Their activity was higher than the starting BC, which is a good candidate for the activation of SPS. Concerning the degradation process, it can be concluded that it takes place through different pathways, including the oxidation of SMX by sulfate and hydroxyl radicals and singlet oxygen. It should be noted that commercial CeO_2_ or CeO_2_-BC calcined at higher temperature was rather inactive. This method is simple and low cost and can be used to obtain hybrid materials with interesting properties.

The application of nanomaterials is not limited only to inorganic compounds; it can be expanded also to polymers. Aromatic polyimides present a variety of excellent physicochemical properties, and they have found significant applications. Polyimides can be easily tuned and different units, such as azobenzene units, can be introduced to their structure. These azo derivatives are optically active and, under the action of linearly polarized light, undergo multiple reversible trans to cis photo-isomerization processes. Moreover, the cyclic photo-isomerization can lead to a large-scale mass transport of the polymer chains, which appear as a surface relief grating (SRG). In the contribution of Iuliana Stoica and Ion Sava group [9], Atomic Force Microscopy (AFM) was used for the evolution of local mechanism and chemical properties under Pulsed UV Laser-Nanoinduced Patterns on Azo-Naphthalene-Based Polyimide Films. Specifically, AFM was applied to determine morphological, statistical, local mechanical, and chemical properties combined with the molecular modeling. Interestingly, the properties were different in various regions due to reorganization of the matter by azo-naphthalene dipoles orientation and trans-cis isomerization of the azo-segments. It was found that polymers with 50% azo groups in cis had either a maximum or a minimum peak of the calculated parameters. Confocal Raman measurements confirm that the cis isomer evolution is mainly responsible for the observed differences.

Medium density fiberboard (MDF) is a natural timber panel, produced from lingo cellulosic fibers and binders under pressure and temperature [10]. MDF can find many applications in furniture industries and interior constructions. MDF has poor physical properties and it cannot be used in moist and hot environments. An option to improve the MDF properties is the addition of multiwall carbon nanotubes (MWCNTs). The embedment of MWCNTs urea formaldehyde resin at concentrations from 0–5% was investigated in [10]. The MWCNTs can deeply penetrate into the wood, effectively altering its surface chemistry and resulting in a high degree of improvement in physical and mechanical strength. It was found that this addition enhanced thermal conductivity by 24.2%, reduced curing time by 20%, and controlled formaldehyde emission by 59.4%. Moreover, properties such as internal bonding, modulus elasticity, modulus of rupture, thickness swelling, and water absorption were significantly improved.
